# CD73 and PD-L1 as Potential Therapeutic Targets in Gallbladder Cancer

**DOI:** 10.3390/ijms23031565

**Published:** 2022-01-29

**Authors:** Lu Cao, Kim R. Bridle, Ritu Shrestha, Prashanth Prithviraj, Darrell H. G. Crawford, Aparna Jayachandran

**Affiliations:** 1 Faculty of Medicine, The University of Queensland, Brisbane, QLD 4120, Australia; l.cao1@uq.edu.au (L.C.); k.bridle@uq.edu.au (K.R.B.); ritu.shrestha@uq.edu.au (R.S.); d.crawford@uq.edu.au (D.H.G.C.); 2 Gallipoli Medical Research Institute, Greenslopes Private Hospital, Brisbane, QLD 4120, Australia; 3 Fiona Elsey Cancer Research Institute, Ballarat, VIC 3350, Australia; prashanth@ballaratoncology.com.au

**Keywords:** gallbladder cancer, cancer stem cells, epithelial-to-mesenchymal transition, CD73, PD-L1, immune checkpoint inhibitors

## Abstract

Gallbladder cancer (GBC) is one of the most common and aggressive biliary tract cancers with a dismal prognosis. Ongoing clinical trials are evaluating a few selected immune checkpoint inhibitors (ICIs) as monotherapy for the treatment of GBC patients. However, only a subset of patients benefits from these treatments. To improve ICI therapy response, molecular mechanisms that confer resistance to immune checkpoint (IC) blockade needs to be explored. Epithelial-to-mesenchymal transition (EMT) program and cancer stem cells (CSCs) have been implicated as key processes that confer ICI treatment resistance. However, in GBC the EMT-CSC-IC axis has not yet been clearly elucidated. This study aims to examine the aberrant expression of ICs associated with CSC and EMT. We successfully enriched CSCs by utilizing a 3-dimensional culture system and established a reversible EMT model with human GBC NOZ cell line. Notably, ICs CD73 and PD-L1 were closely associated with both CSC and EMT phenotypes. Knockdown of CD73 or PD-L1 reduced the proliferative and motile abilities of both adherent monolayers and anchorage-free spheroids. In conclusion, blocking CD73 and PD-L1 offer a promising therapeutic strategy for targeting highly aggressive populations with CSC and EMT phenotype to improve GBC patient prognosis.

## 1. Introduction

Gallbladder cancer (GBC) is a rare but highly lethal cancer of the biliary tract system with estimated 5-year overall survival rates of 19% for all stages jointly analyzed and 2% in a metastatic setting [1]. According to GLOBCAN, whilst 1.2% new cases of GBC are diagnosed annually, GBC accounts for 1.7% of all cancer deaths worldwide [2,3,4]. The extremely poor prognosis of GBC is ascribed to the prolonged asymptomatic early stage with atypical clinical symptoms, late diagnosis with unresectable or metastatic disease, and suboptimal treatment modalities [5]. Standard first-line treatment options including chemotherapy and radiotherapy confer modest survival benefit for locally advanced and metastatic stages of GBC [6]. Furthermore, currently no immune checkpoint inhibitors (ICIs) or targeted therapies against actionable mutations have been approved for treatment of GBCs. Thus, there is a pressing need for exploring novel and better treatment strategies to improve survival in GBC patients. 

GBC is characterised by high intracellular heterogeneity that represents a substantial obstacle to increasing effective response to therapies in patients. Cancer stem cells (CSCs) are minor subpopulation of tumor cells that are responsible for maintaining cellular heterogeneity, cancer sustaining growth, metastasis, and drug resistance [7,8]. This highlights the importance of developing refined treatment strategies to eradicate CSCs to yield higher treatment efficacy. Whilst the identification and characterisation of cancer stem-like cells in other solid tumors have been widely investigated, there are only a few studies reporting the isolation and in vitro culture of stem-like populations in GBC. Manohar et al. identified EpCAM^+^CD13^+^CD44^+^ cells in the human primary fetal gallbladder that were capable of single cell self-renewal, long-term expansion in vitro and were able to engraft in vivo, implicating EpCAM, CD13, and CD44 as CSC markers in GBC [9]. Furthermore, CSC markers OCT4, CD133, and EpCAM were positively associated with tumor grading, staging and metastasis and/or survival in GBC patients [10,11]. These unique set of molecules together with other CSC and pluripotent stem cell markers routinely utilised for the isolation and characterisation of CSCs in other solid tumors have been used to distinguish gallbladder CSCs from the tumor bulk cells [12,13]. 

Epithelial-to-mesenchymal transition (EMT), another fundamental hallmark of cancer progression, facilitates cancer intracellular heterogeneity [14]. EMT is a cellular reprograming process whereby a polarised epithelial cell transits into a motile mesenchymal cell, enhancing the migratory and invasive potential of tumor cells. Conversely, a mesenchymal cell can regain epithelial state by undergoing mesenchymal-to-epithelial transition (MET) to facilitate tumor recurrence [15]. Loss of epithelial markers, such as E-cadherin and occludin, and upregulation of mesenchymal markers—including N-cadherin, Vimentin, Fibronectin, Zeb, Snail, and Slug—are utilised to monitor the EMT process [15]. Multiple studies have demonstrated that EMT is closely correlated with GBC progression and prognosis in the clinic [16]. Recent evidence showed a direct link between the EMT state and the gain of CSC properties [17,18,19,20,21]. Both CSCs and EMT contribute to drug resistance in cancers and tumor cells exhibiting both stemness and EMT traits have increased potential for invasion and metastasis [22,23]. As CSCs and EMT provide an underlying mechanistic basis for tumor metastasis, cancer relapse, and drug resistance, their study may provide new insights into developing effective anti-tumor strategies in GBC.

ICI-based immunotherapy that effectively reanimates the T-cell associated antitumor response has set a new benchmark for improving the treatment of many malignant cancers in recent years [24,25]. As immune system dysregulation is associated with the pathogenesis of GBC, ICIs offer an attractive treatment option [26,27]. However, the knowledge about immune modulators in the therapeutic context remains very limited. Ongoing clinical trials in advanced biliary tract cancer patients reported that patients responded to ICI monotherapies with anti-programmed cell death ligand-1 (PD-L1), anti-programmed cell death protein-1 (PD-1), and anti-cytotoxic T lymphocyte-associated antigen-4 (CTLA-4) [28]. However, a large portion of patients have only temporary or no response to this immunotherapeutic approach, the mechanisms of resistance need to be understood. Previously, we have reported that CSCs and EMT are intrinsically associated with the expression and regulation of immune checkpoints (ICs) in hepatocellular carcinoma and cholangiocarcinoma [12,13]. Due to the vital role of CSCs and EMT in GBC therapy resistance, cancer progression, and relapse, it is of interest to investigate immune modulatory molecules that are differentially expressed in more aggressive subpopulations of CSCs and/or cells undergoing EMT compared to other tumor cells comprising bulk of the tumor. There is a knowledge gap about the CSC-IC-EMT axis in GBC. The aim of this research was to identify additional potential candidates for immunotherapeutic treatment by exploring the association between CSC, EMT, and ICs in GBC. To this end, we enriched cancer stem-like cells in a non-adherent culture system and established a reversible EMT induction model in vitro to examine the distribution and regulation of a panel of immune checkpoints in these cells.

## 2. Results

### 2.1. Anchorage-Independent 3D GBC Spheres Express Embryonic Stemness and CSC Markers

**Figure 1:**
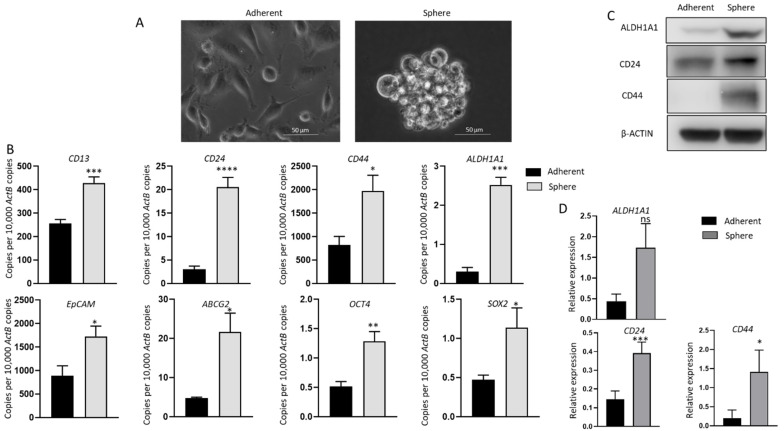
Anchorage-independent 3-dimensional spheroid culture enriches CSC population in NOZ cells. (**A**) Monolayer culture and 3-D culture of NOZ cells. (**B**) Reverse transcription-quantitative (RT-q) PCR analysis demonstrated higher expression of embryonic stemness and CSC markers in NOZ spheres compared to adherent monolayers. (**C**,**D**) Western blots demonstrated the upregulation of ALDH1A1, CD24, and CD44 in NOZ spheres. * *p* < 0.05, ** *p* < 0.01, *** *p* < 0.005, **** *p* < 0.001, ns—not significant.

To assess the relationship between immune modulators and CSCs, the human GBC cell line NOZ was cultured in a serum-free stem cell-conditioned medium to generate non-adherent 3D sphere clusters. Sphere culture has been increasingly used as a tool for enriching stem cells which relies on their nature of anchorage independent growth in serum deprived conditions [12,13,29,30,31]. Figure 1A shows NOZ cells grew as adherent monolayer cells in serum-containing medium and formed anchorage-independent spheres in serum-free conditioned medium on day 7. The stemness of spheres was corroborated by examining the expression of stem cell-associated markers by RT-qPCR or/and Western blot analyses. The stemness markers *CD13*, *CD24*, *CD44*, *ALDH1A1*, *EpCAM*, ATP-binding cassette transporter *ABCG2* and pluripotency-related genes *OCT4* and *SOX2*, which have been commonly used to identify CSCs in gastrointestinal cancers, were remarkably upregulated in NOZ spheres compared with adherent parental monolayer cells by RT-qPCR (Figure 1B). The higher expression levels of ALDH1A1, CD24, and CD44 in spheres were also validated by Western blot (Figure 1C,D). These findings indicate that the 3D culture system in the serum-free formula can selectively enrich cancer stem-like cells from NOZ cell line; therefore, the resulting spheres can be used for further CSC-orientated studies.

### 2.2. Increased Expression of Immune Modulators in GBC Derived Stem-Like Cells

**Figure 2:**
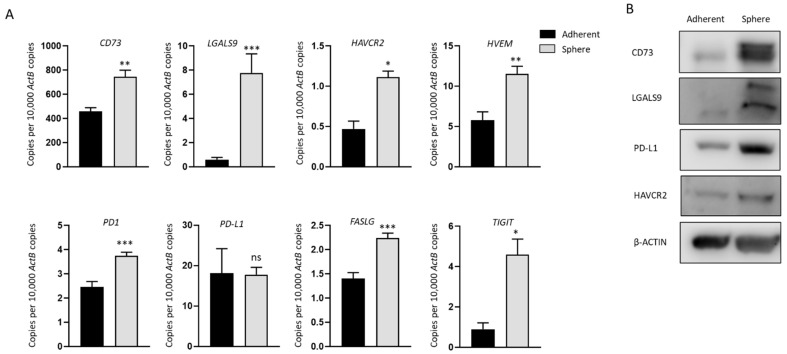
Immune modulators are upregulated in NOZ stem-like population. (**A**) RT-qPCR showed immune modulators *CD73*, *LGALS9*, *HAVCR2, HVEM*, *PD1*, *PD-L1*, *FASLG*, and *TIGIT* were upregulated in NOZ spheres compared to its parental adherent cells. (**B**) Western blots demonstrated the increase of CD73, LGALS9, PD-L1, and HAVCR2 in NOZ spheres. * *p* < 0.05, ** *p* < 0.01, *** *p* < 0.005, ns—not significant.

We analyzed the expression of a panel of immune modulators in NOZ cells cultured as adherent monolayers and in 3D condition. These immune regulators were chosen as we have previously demonstrated their associated with a poor prognosis in patients with hepatocellular carcinoma and cholangiocarcinoma [12,32]. mRNA levels of *CD73 (NT5E), LGALS9 (Galectin-9), HAVCR2 (TIM-3), HVEM (TNFRSF14), PD1, FASLG*, and *TIGIT* were significantly increased in spheres (Figure 2A), in line with the elevated expression of CD73, LGALS9, and HAVCR2 at the protein level compared with adherent cells (Figure 2B). Whilst no change in *PD-L1* expression was detected by RT-qPCR between spheres and adherent cells, upregulation of PD-L1 in spheres was detected by Western blot (Figure 2B). Given the higher expression of immune modulators in stem-like cells, these cells may be more susceptible to ICI-based immunotherapy. 

### 2.3. Expression of CSC Markers and Immune Modulators Varied from Tumor and Para-Tissues in GBC Patients

**Figure 3:**
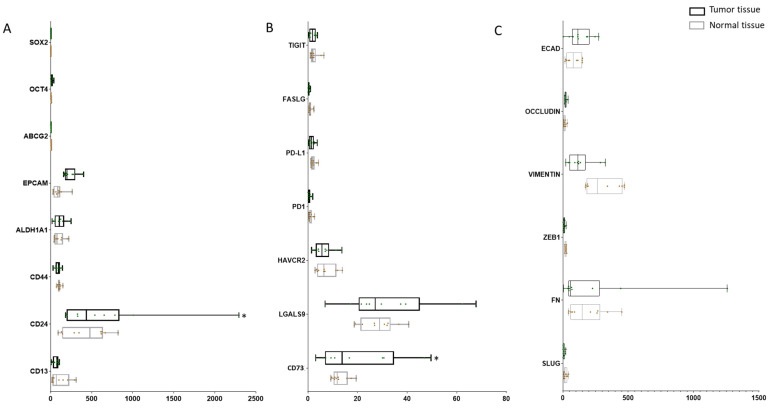
Expression of CSC markers and immune modulators varied from tumor and para-tissues in GBC patients. (**A**) Expression level of CSC markers *SOX2, OCT4, ABCG2, EPCAM, ALDH1A1, CD44, CD24*, and *CD13* in matched tumor and para-tumor tissues in GBC patients. (**B**) Expression level of immune checkpoints *TIGIT, FASLG, PD-L1, PD1, HAVCR2, LGALS9*, and *NT5E* in matched tumor and para-tumor tissues in GBC patients. (**C**) Expression level of EMT markers *ECAD*, *OCCLUDIN*, *VIMENTIN*, *ZEB1*, *FN*, and *SLUG* in matched tumor and para-tumor tissues in GBC patients. * *p* < 0.05.

To investigate the expression of CSC markers, ICs, and EMT markers in GBC patients, we accessed the GEO sequencing dataset which includes 10 cases of GBC tumorous tissue with matched para-tumor tissue and analyzed the expression levels of CSC, IC, and EMT genes. A panel of CSC markers *SOX2*, *OCT4*, *ABCG2*, *EPCAM*, *ALDH1A1*, *CD44*, *CD24*, and *CD13* showed varied expression across tumor and normal tissues, with significantly higher expression of *CD24* in tumor compared with normal tissue (*p* = 0.008). With regard to ICs, the expression of *HAVCR* (*p* = 0.782), *LGALS9* (*p* = 0.179) and *NT5E* (*p* = 0.015) were higher in tumor tissue in comparison with para-tumor. No significant changes in EMT marker expression was noted across tumor and normal tissues (Figure 3). 

### 2.4. TGF-β1 Induces a Reversible EMT in GBC 

**Figure 4:**
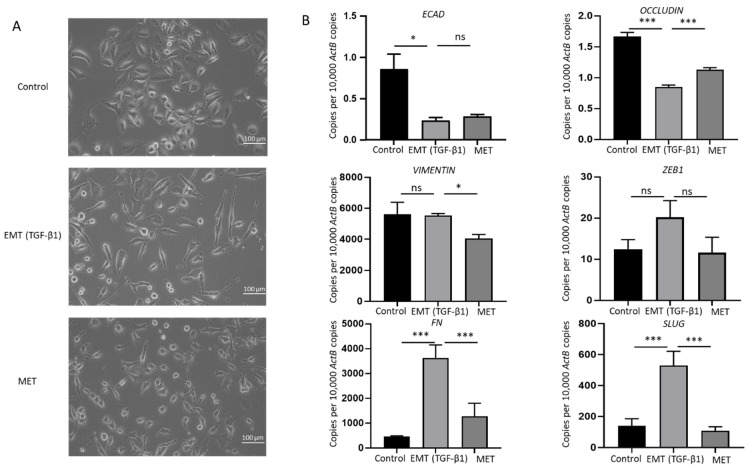
TGF-β1 induces reversible epithelial to mesenchymal transition (EMT) in NOZ cells. (**A**) Representative images of NOZ cells before and after incubating with TGF-β1 and after withdrawal of TGF-β1 under an inverted phase contrast microscope at ×200 magnification. (**B**) RT-qPCR showed TGF-β1 decreased expression of *ECAD* and *OCCLUDIN* and increased expression of *VIMENTIN*, *ZEB1*, *FN*, and *SLUG*. Withdrawal of TGF-β1 induced MET consistent with elevated expression of *ECAD* and *OCCLUDIN* and decreased expression of *VIMENTIN*, *ZEB1*, *FN*, and *SLUG*. * *p* < 0.05, *** *p* < 0.005, ns—not significant.

**Figure 5:**
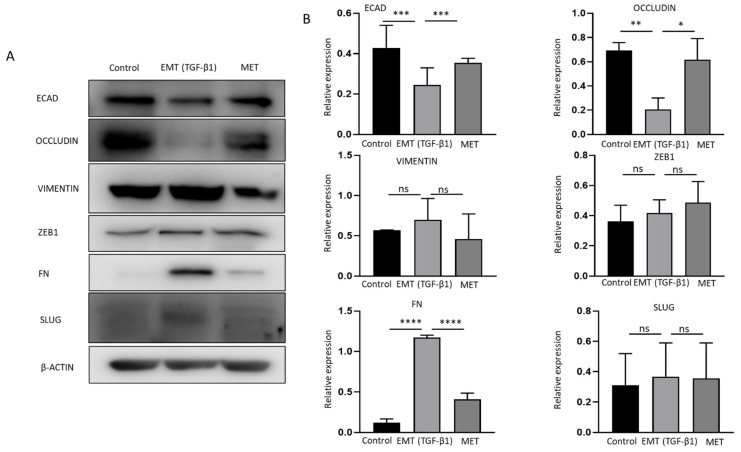
Reversible epithelial to mesenchymal transition (EMT) is mediated by TGF-β1 in NOZ cells. (**A**,**B**) Western blot analysis showed TGF-β1 decreased expression of ECAD and OCCLUDIN and increased expression of VIMENTIN, ZEB1, FN, SLUG. Withdrawal of TGF-β1 induced MET consistent with elevated expression of ECAD and OCCLUDIN and decreased expression of VIMENTIN, ZEB1, FN, and SLUG. * *p* < 0.05, ** *p* < 0.01, *** *p* < 0.005, **** *p* < 0.001, ns—not significant.

Cytokines transforming growth factor (TGF)-β1 and tumor necrosis factor (TNF)-α are potent inducers of EMT in many cancers [33,34]. To evaluate the association between EMT and immune checkpoint molecules, NOZ cells were incubated with 10 or 20 ng/mL of TGF-β1 and TNF-α separately for 96 h. NOZ cells were more responsive to TGF-β1 than TNF-α (Figure S1). 10 and 20 ng/mL of TGF-β1 induced a robust EMT with decreased expression levels of epithelial markers *E-cadherin, Occludin*, and *CK19*, and increased expression levels of mesenchymal markers *N-cadherin, SLUG, SNAIL, ZEB1*, and *Fibronectin (FN)* (Figure S1). Therefore, TGF-β1 at a concentration of 10 ng/mL was selected for the following EMT induction study. Morphological alterations due to TGF-β1 treatment and withdrawal were observed under a bright-field microscope. Figure 4A images showed a disruption of cell-to-cell junction and acquisition of elongated spindle-shaped morphology in the cells post TGF-β1 induction for 72 h and subsequent restoration to compact cell phenotype after withdrawal of TGF-β1 for another 72 h. The induction of EMT was evidenced by qRT-PCR with a decrease in epithelial markers (E-cadherin and occludin), accompanied by an increase in mesenchymal markers (vimentin, ZEB1, FN, and SLUG). MET was confirmed by reversed changes of these markers after removing TGF-β1 (Figure 4B). Induction of EMT followed by MET changes associated with TGF-b1 treatment was confirmed at the protein level by Western blotting (Figure 5A,B). This reversal model was then used for investigating the association of immune checkpoint molecules and EMT.

### 2.5. Reversal of TGF-β1-Mediated EMT Reverses the Expression of Immune Checkpoints CD73 and PD-L1

**Figure 6:**
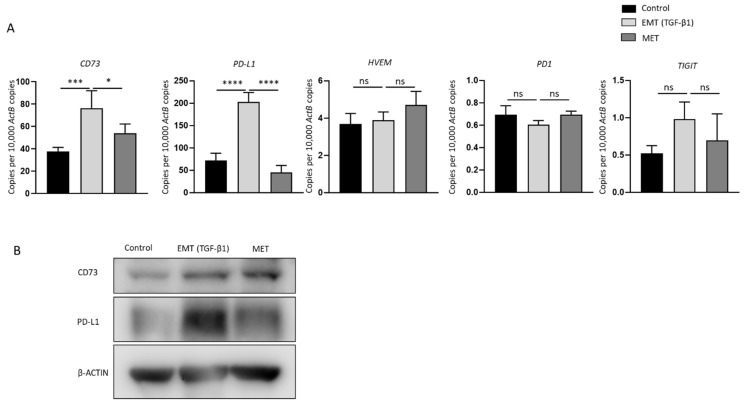
Expression of immune checkpoints CD73 and PD-L1 correlates with EMT-MET status of NOZ cells. (**A**) RT-qPCR demonstrated upregulation of immune checkpoint molecules *CD73* and *PD-L1* in EMT-induced NOZ cell. A reduction in expression of *CD73* and *PD-L1* was observed following reversal assay. No significant change in the expression of *HVEM*, *PD1*, and *TIGIT* was noted during EMT or MET. (**B**) Protein expression level of CD73 and PD-L1 as observed by Western blot matched mRNA expression level. * *p* < 0.05, *** *p* < 0.005, **** *p* < 0.001, ns—not significant.

TGF-β1-induced EMT and MET transformed cells were evaluated for expression of immune modulators including *PD-L1*, *CD73*, *HVEM*, *PD1*, and *TIGIT* that were closely related with CSC status in NOZ cells. The RT-qPCR results revealed that *CD73* and *PD-L1* were remarkably upregulated with the treatment of TGF-β1 and downregulated upon the removal of TGF-β1. However, *HVEM*, *PD1,* and *TIGIT* expression was not altered during the changes of cellular epithelial and mesenchymal states (Figure 6A). The reversible alteration of CD73 and PD-L1 was also confirmed by Western blot analyses (Figure 6B). These results suggest that EMT is closely involved in the regulation of immune checkpoints CD73 and PD-L1 expression. Thus, CD73 and PD-L1-targeted therapies may favour eliminating the aggressive population of cancer cells that are undergoing EMT.

### 2.6. CD73 and PD-L1 Knockdown Inhibits Cell Growth and Reduces Cell Motility

**Figure 7:**
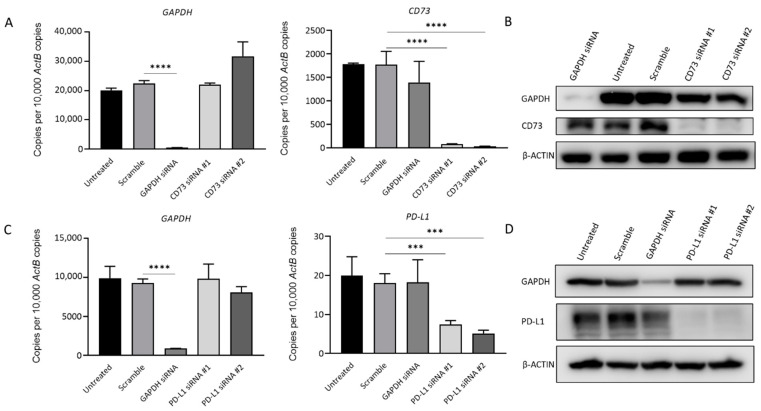
siRNA-mediated knockdown assay effectively represses CD73 and PD-L1 expression in NOZ cells. NOZ cells were transfected with CD73 or PD-L1 siRNA, untransfected, scrambled siRNA, and GAPDH siRNA as controls. Effective knockdown of CD73 was detected by RT-qPCR (**A**) and Western blot (**B**). PD-L1 expression significantly reduced following treatment with PD-L1 specific siRNAs as detected by RT-qPCR (**C**) and Western blot (**D**). *** *p* < 0.005, **** *p* < 0.001.

To further evaluate the impact of CD73 and PD-L1 on cell growth, and migration in GBC, siRNA knockdown assays were performed. Figure 7 shows the transfection with specific siRNAs efficiently reduced CD73 and PD-L1 expression separately in NOZ cells at the mRNA and protein level (Figure 7A–D). 

**Figure 8:**
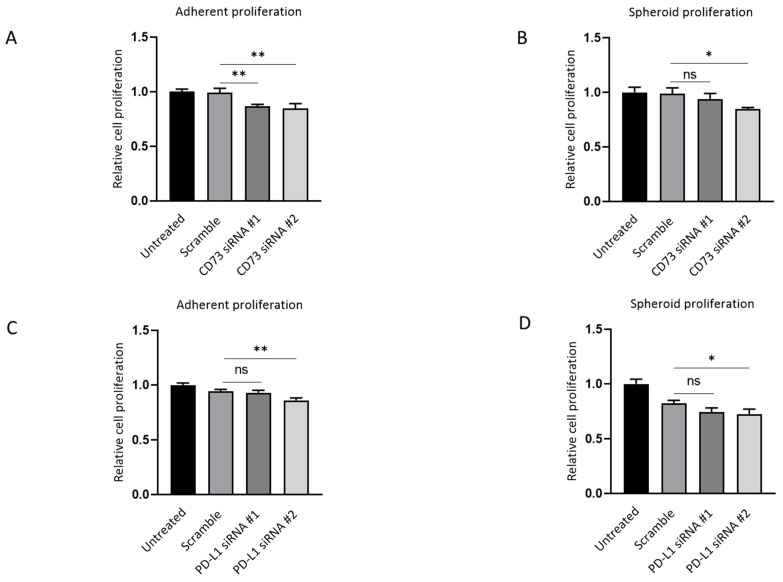
CD73 and PD-L1 is involved in proliferation of 3D spheres and adherent monolayer NOZ cells. MTS assays indicated CD73 knockdown inhibited cell growth both in monolayer adherent condition (**A**) and in anchorage-independent 3D condition (**B**) MTS assays demonstrated that PD-L1 knockdown suppressed cell proliferation both in monolayer adherent condition (**C**) and in anchorage-independent 3D condition (**D**). * *p* < 0.05, ** *p* < 0.01, ns—not significant.

Next, the effect of depleting CD73 and PD-L1 on cell growth in both adherent monolayers and spheroids was evaluated. CD73 knockdown resulted in ~15% inhibition of both adherent and spheroid proliferation compared with the scrambled siRNA control, as assessed by MTS assay (Figure 8A,B). CD73 siRNA #1-treated cells did not show significant suppression in spheroid proliferation compared with the scrambled control (Figure 7B). PD-L1 #2 knockdown led to ~10% repression of adherent and spheroid proliferation as compared with the scramble (Figure 8C,D).

**Figure 9:**
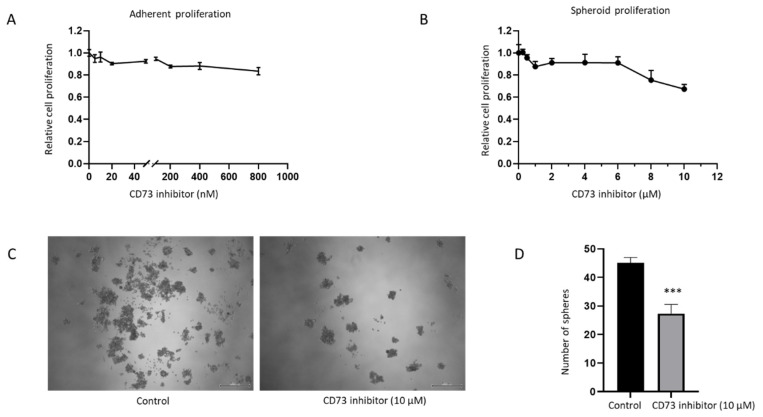
NOZ cell line was not sensitive to CD73 inhibitor PSB 12379 at the physiological concentration range. MTS assays indicated no reduced proliferation of adherent monolayer cells upon CD73 inhibitor treatment (**A**). Higher doses of CD73 inhibitor reduced proliferation of anchor-age-independent 3D spheres (**B**). Photomicrographs of NOZ spheres on day 7 treated with or without 10 µM CD73 inhibitor (**C**). NOZ spheres numbers were enumerated in control and CD73 inhibitor treatment conditions (**D**). *** *p* < 0.005.

The effect of CD73 depletion on cell growth in both adherent monolayers and spheroids was assessed with a pharmacological CD73 inhibitor, PSB 12379. PSB 12379 is a selective high affinity CD73 inhibitor that blocks ecto-5’-nucleotidase-mediated adenosine production by preventing the conversion of AMP to adenosine [35]. Both the adherent monolayer and spheroids were treated at increasing concentrations of CD73 inhibitor for 72 h. However, no effect on proliferation was observed with this inhibitor on the adherent monolayer or spheroids at Ki value range for inhibitor activity from 2.21 to 9.03 nM (Figure 9A,B). At higher concentration of 10 µM of the inhibitor, we observed reduced proliferation of spheroids and lesser number of spheres following treatment (Figure 9B–D). This sensitivity may be due to toxicity at high dose of the inhibitor. Given that NOZ cell line was not sensitive to PSB 12379 inhibitor within the physiological concentration range, we utilised specific siRNA targeting CD73 for further assessments.

**Figure 10:**
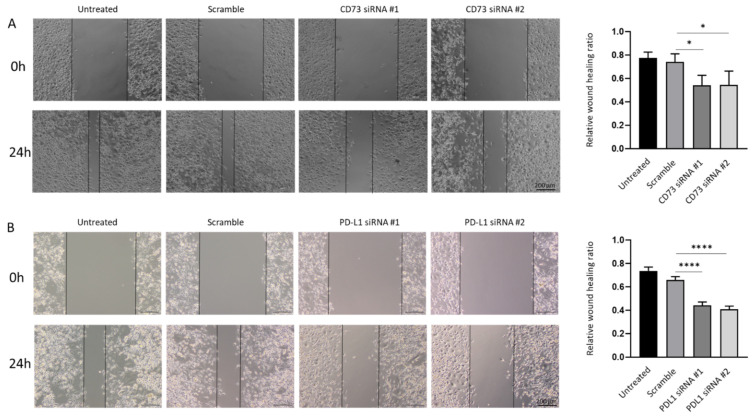
CD73 and PD-L1 is involved in wound healing ability of NOZ cells. Scar healing assay (24 h) demonstrated CD73 (**A**) and PD-L1 (**B**) knockdown slowed wound healing as compared to control cells. Bar charts show the ratio of the reduction of the scar length compared to the initial scar length (×200 magnification). * *p* < 0.05, **** *p* < 0.001.

As increased migratory ability is an important feature associated with cancer metastasis, we further examined whether the repression of CD73 and PD-L1 reduces motile behaviour associated with GBC cells. Quantification of the open wound area showed the CD73 and PD-L1 depleted cells migrated slower from the edges of the scratch as compared with those in untreated and scrambled siRNA control groups (Figure 10A,B). 

**Figure 11:**
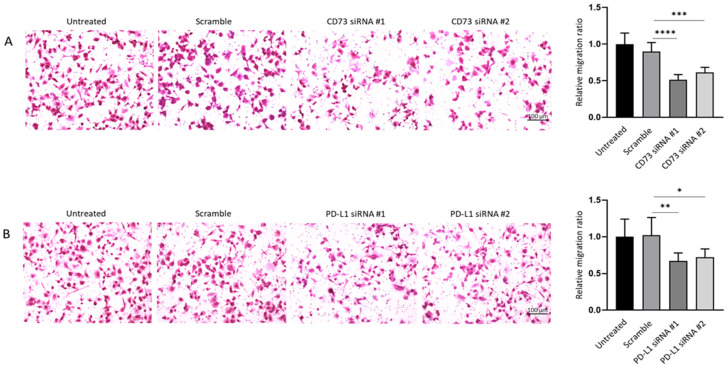
CD73 and PD-L1 is involved in migration ability of NOZ cells. Transwell assays (24 h) showed CD73 (**A**) and PD-L1 (**B**) knockdown reduced cell migration compared to control cells. Bar charts show the ratio of migrated cells normalised with that of the untransfected group (×200 magnification). * *p* < 0.05, ** *p* < 0.01, *** *p* < 0.005, **** *p* < 0.001.

Similarly, crystal violet staining on the lower surface of the Transwell membrane showed the knockdown of CD73 and PD-L1 led to a notable decrease in the number of migrating cells in siRNA treatment groups as compared with the control groups (Figure 11A,B). Therefore, wound healing and Transwell assays demonstrated that both CD73 and PD-L1 knockdown notably reduced proliferative and migratory capacities of NOZ cell line.

## 3. Discussion

With the ICIs opening a new arena of targeted therapy in a number of solid neoplasms including liver cancers, novel ICI-based immunotherapies may yield promising responses in GBC patients. Currently, a few ICIs against PD-1, PD-L1, and CTLA-4 are under exploration in GBCs at different clinical stages either alone or in combination with other immunotherapy agents or/and chemotherapy [28]. Furthermore, new ICs such as VISTA, TIM-3, LAG3, and CD73 have been identified and are under investigation [36,37,38,39], which broadens the potential for immunotherapy in the treatment of several malignancies. However, there are very limited studies evaluating ICs in GBC.

In this study, we enriched stem-like cells from GBC NOZ cells using anchorage-independent 3D sphere culture, and then examined a panel of immune modulators. A number of immune modulators displayed higher expression levels in NOZ stem-like cells including CD73, PD-L1, LGALS9, HAVCR2, HVEM, PD1, FASLG, and TIGIT, as compared with their parental adherent cells. Notably, we have demonstrated two immune checkpoints—CD73 and PD-L1 were also closely related to EMT status, evidenced by elevated expression of CD73 and PD-L1 expression upon TGFβ-1-facilitated EMT and the reversal (MET) attenuated their expression levels. Given that CSCs and EMT phenotypes are the two cellular fractions that are most associated with aggressiveness, cellular heterogeneity, metastasis and therapy resistance of cancer, it raises the importance of CD73 and PD-L1 as potential promising drug targets in GBC.

CD73 (NT5E), an ecto-5′-nucleotidase, acting as an inhibitory immune checkpoint molecule, generates adenosine from ATP in tandem with CD39 (ecto-ATPase). Adenosine potently impairs antitumor immunity by suppressing T-cell activation, tumor cell killing of cytotoxic T lymphocytes (CTL), natural killer (NK) cell, and lymphokine-activated killer (LAK) cell function [40]. CD73 has emerged as an attractive target for cancer treatment to improve anti-tumor immunity within the last decade and some phase I/II clinical trials in different types of cancers are under investigation [41]. However, the available evidence in GBC is limited. The present study is the first to correlate CD73 with both CSC and EMT states in GBC. It has been reported that CD73 is highly expressed in many solid tumors and promotes tumor cell proliferation and motility in gastric, cervical, and pancreatic cancers [42,43,44]. High expression of CD73 has been associated with poor prognosis in HCC, pancreatic, colorectal, and breast cancers [32,44,45,46,47]. CD73 has been linked with stemness in gastric cancer [48]. Besides, high CD73 expression is correlated to EMT in HCC and cholangiocarcinoma [12,49]. Gene expression profiling of a TGF-β1 induced GBC cell line GBC-SD revealed CD73 as the most upregulated gene among 225 differentially expressed genes [50]. We found that treatment with CD73 inhibitor PSB 12379 at the recommended physiological dose range of 2.21 to 9.03 nM showed no effect on the proliferation rates of both NOZ adherent monolayer and spheroids. Interestingly, at higher concentrations of CD73, inhibitor spheroid proliferation and number of spheroids were reduced. Others have reported similar findings using another CD73 inhibitor, 12 µM α,β-methylene adenosine 5’-diphosphate (APCP). For instance, APCP inhibited growth of breast cancer cells MDA-MB-231 at a low concentration range of 3 to 12 µM, while a higher concentration of the inhibitor ranging from 100 to 500 µM was required to block proliferation in glioma cells [51,52]. These differences in doses utilised may be ascribed to different cell lines used. Further studies are required to investigate CD73 catalytic activity with more potential selective inhibitors in GBC cells. PD-L1 small molecular pharmacological inhibitors are in the developmental phase and examining their effect on NOZ adherent monolayer and spheroids is warranted [53]. Further studies with monoclonal antibodies targeting CD73 and PD-L1 can be tested to corroborate the findings from this study with siRNAs.

Consistent with our findings, knockdown of CD73 inhibited cell proliferation, invasion, and migration. In addition, CD73 has been shown to function as a pivotal downstream mediator of miR-30b/miR-340 in GBC [54]. The current study is the first to highlight the overexpression of CD73 in GBC stem-like cells. CD73 has been demonstrated to be a regulator of stemness and EMT in ovarian cancer and inhibition of CD73 reduced sphere formation and tumorigenesis [55]. A study showed that in a cohort of 108 gallbladder adenocarcinoma patients, CD73 can function as an independent biomarker for assessing disease progression and prognosis [50]. Further studies using in vivo models are needed to examine the possible therapeutic benefit of targeting CD73 in GBC.

As the first and the most extensively documented immunotherapeutic target, a tumor-intrinsic role of PD-L1 in modulating EMT and CSC has been discovered in some solid cancer types [56,57]. The correlation of PD-L1 in GBC has been promising. A study in 101 GBC patient tissues indicated that PD-L1 expression level was significantly associated with poor clinicopathological parameters, overall survival, and progression-free survival [58]. This contrasts with another study showing that PD-L1 expression had no significant association with either tumor size or overall survival in GBC patients, although PD-L1 was expressed in 23% of tumor samples (*n* = 174) [59]. Similarly, Mody et al. reported PD-L1 expression was noted in 12.3% (25/203) of tumor samples [60]. Abdel-Wahab et al. found tumor PD-L1 expression was positive in 15.6% (119/790) GBC patients [61], and another study reported a 54% PD-L1 positivity in GBC patients (*n* = 66), which included 18% in tumor cells and 36% in peritumoral immune stroma [62]. Given that PD-L1 expression in tumor cells has been associated with increased clinical benefit from PD-1 inhibition in some tumor types [4,63,64], these findings in GBC support the future possibility of PD-L1 targeted therapy in PD-L1 positive GBC patient subset. Promisingly, a case report has reported a recurrent metastatic GBC patient with strong PD-L1 expression obtained a remarkable response to radiotherapy in combination with nivolumab, a PD-1 antibody treatment [65]. Our observation of a striking association of PD-L1 with stemness and EMT in the present study highlights the importance of PD-L1 as a therapeutic target in GBC.

Furthermore, we envisage that monotherapies with agents targeting CD73 or PD-L1 may not be effective in eliminating CSCs based on our observation that CD73 or PD-L1 knockdown resulted in 15% or 10% inhibition of both adherent and spheroid proliferation. Thus, further investigation into whether combination treatment including EMT inhibitors and/or CSC inhibitors with CD73 or PD-L1 blockade may result in the effective elimination of both GBC CSCs and tumor bulk must be examined. A recent study highlighted a role for metabolic adaptations including enhanced glycolysis, oxidoreductase activity, and mitochondrial oxidative phosphorylation that facilitate the development of resistance to checkpoint blockade therapy [66]. Thus, agents that can rewire metabolic pathways may be an alternative approach to combine with either CD73 or PD-L1 treatment in GBC.

Our study has some important limitations. We have examined the NOZ cell line in this study and corroboration of these findings in additional GBC cell lines and primary cells is warranted. Our study examined enrichment of CSC markers by RT-qPCR and Western blotting. Other techniques including flow cytometry and immunofluorescence for examining localisation changes in CSC markers in an intact cell will be informative. We were unable to evaluate the association of IC-EMT-CSC axis to GBC patient prognosis as the GBC patients evaluated in this study lacked clinical and survival data. Thus, further studies are needed to assess the link between IC-EMT-CSC axis and prognosis in larger cohorts of GBC patients. Moreover, further investigation into the molecular mechanisms underpinning the regulating CD73 and PD-L1 is required both in vitro and in vivo settings to enable development of efficacious and durable therapies. Given that the CSC-EMT-IC axes are crucial players in the development of resistance to radiation and cytotoxic agents, future studies are required to examine the specific role CD73 and PD-L1 in the development of radio- and chemo- resistance in GBCs. Furthermore, CSCs are characterised by their unique capability to maintain self-renewal [67]. Further studies will be required to determine whether the self-renewal capacity of GBC CSCs can be impacted by targeting CD73 and/or PD-L1 expression.

Taken together, CD73 and PD-L1 may be central players in CSC and EMT-related oncogenesis. Thus, the blockade of CD73 and PD-L1 may serve to eliminate the aggressive cell subsets with CSC and EMT traits and therefore may be promising targets in strategies to improve the efficacy of GBC treatment. 

## 4. Materials and Methods

### 4.1. Cell Culture

The human GBC cell line NOZ was obtained from Prof. John Mariadason, Olivia Newton-John Cancer Research Institute, Australia. Cells were cultured in DMEM with 10% FBS (Gibco) and 1% penicillin/streptomycin (Gibco) and maintained in a humidified incubator at 37 °C with 5% CO_2_. 

### 4.2. Sphere Culture

Sphere culture was performed as previously reported [12]. Briefly, monolayer cells were detached by trypsin-EDTA and washed to remove serum, and then suspended in serum-free DMEM/F12 medium supplemented with 20 ng/mL recombinant human epidermal growth factor (EGF) (Peprotech), 10 ng/mL recombinant human fibroblast growth factor (FGF) (Peprotech), 2% B27 supplement without vitamin A (Gibco), and 1% N2 supplement (Gibco). The cells were subsequently seeded in ultra-low attachment 6-well plates (Corning) at a density of 5000 cells/well. The spheres were maintained in a humidified incubator at 37 °C with 5% CO_2_ for 7 days and then collected by gentle centrifugation. Dissociation was performed by trypsin-EDTA with gentle mechanical disruption by pipetting. To passage, the resulting single cells were centrifuged to remove trypsin and re-suspended in the serum-free medium, allowing spheres to re-form. 

### 4.3. RNA Extraction, cDNA Synthesis, and RT-qPCR 

RNA extraction, cDNA synthesis and RT-qPCR were performed as previously described [68]. Briefly, total RNA was extracted using Bioline Isolate II RNA Mini Kit and 1 μg RNA was then taken for reverse transcription using Bioline SensiFAST cDNA Synthesis Kit, according to manufacturer’s instructions. qRT-PCR was performed using Lo-ROX SYBR Green (Bioline) on a ViiA7 Applied Biosystems Real-Time PCR system following a three-step cycle procedure: 95 °C for 2 min, 63 °C for 20 s and 75 °C for 20 s. *β-actin* was used as an internal control. The primers used were previously reported [12] and other primers are listed in Table S1. Expression level is presented as copies of the target gene per 10,000 copies of *β*-actin. 

### 4.4. Protein Preparation and Western Blotting Analysis

Cell extracts from adherent and spheres were harvest on ice using RIPA buffer (Thermofisher) supplemented with complete protease inhibitors (Roche) and phosSTOP phosphatase inhibitors (Roche). The total protein concentration was determined with a Pierce BCA protein assay kit (Thermofisher). Proteins extracts (10 µg) were fractionated by SDS-PAGE and transferred to polyvinylidene difluoride film (PVDF) membranes (Millipore). After blocking with 5% skim milk the membranes were incubated with specific primary antibody at 4 °C overnight and followed by incubation with the horseradish peroxidase (HRP) conjugated secondary antibody. Blots were developed using the chemiluminescence reagent, SuperSignal West Femto Maximum Sensitivity Substrate (Thermofisher), and visualised with Image Quant LAS 500 detection system. Images were quantified with Image Studio™ Lite v5.2 software (LI-COR Biosciences). *β*-actin was selected as a loading control. Antibodies used in this study were previously reported [12] and the others are listed in Table S2. Figures S2–S7 include all original Western blots images of the main figures. 

### 4.5. EMT and MET Reversible Assay

EMT induction assays were performed by incubating cells with indicated doses of TGF-β1 (Peprotech) or TNF-α (Peprotech) in the culture medium. For the reversal of EMT, the medium was completely removed, and cells washed with PBS thoroughly. Cells were then incubated without an EMT inducer for another 3 days.

### 4.6. Expression of CSC Markers and Immune Checkpoint Molecules in GBC Patients

Expression profiling by next-generation sequencing data was accessed through NCBI by GSE139682 (https://www.ncbi.nlm.nih.gov/geo/, accessed on 21 September 2021). The data was used to generate a graphical summary of gene expression changes in CSC markers and immune checkpoint molecules across 10 cases of human GBC para-tumor and tumor samples. Multiple unpaired *t*-test analysis method was used to evaluate the gene expression changes. 

### 4.7. siRNA and Knockdown

For CD73 knockdown, two siRNA sequences specifically targeting human CD73 (s9734 and s9735) were purchased from Thermofisher. For PD-L1 knockdown, two siRNA sequences targeting human PD-L1 (s26547 and s26548) from Thermofisher were used. Control cells were untransfected or transfected with a non-specific scrambled siRNA, or a siRNA sequence targeting GAPDH. NOZ cells were cultured overnight and transfected with siRNA using Lipfectamine RNAimax (Invitrogen) as instructed by the manufacturer. Cells were collected 72 h after transfection for assessment of knockdown efficiency and seeded for proliferation and motility experiments. 

### 4.8. Cell and Sphere Proliferation Assay

The proliferation of cells was measured by CellTiter 96^®^ AQueous One Solution Cell Proliferation Assay Kit (MTS) (Promega). Briefly, non-transfected, scrambled siRNA, PD-L1, and CD73 siRNA transfected cells were seeded in 96-well plates and incubated for designated periods, the MTS reagent was then added to each well according to the manufacturer’s instructions. Absorbance was measured at 490 nm. For spheroid proliferation, cells were cultured in serum-free stem cell-conditioned medium as described above.

### 4.9. CD73 Inhibitor Treatment Assay 

Adherent and spheroid NOZ cells were seeded in 96-well plates and were exposed for 72 h to PSB 12379 (CD73 pharmacological inhibitor) (Tocris) at indicated doses. The MTS reagent was then added to each well and absorbance was measured at 490 nm. Enumeration of spheroids was performed by initially seeding spheroids at 2000 cells/well in a 6-well ultra-low-attachment plates (Corning Incorporated). Spheroids were cultured with serum-free stem cell-conditioned medium. PSB 12379 was added at a concentration of 10 µM to the spheroid culture. Number of spheres per culture well were counted on day 7 using an inverted microscope equipped with a digital camera (Olympus DP21).

### 4.10. Wound Healing Assay

NOZ cells transfected with CD73, PD-L1, or scrambled siRNAs and non-transfected cells were collected and re-seeded in a 12-well plate in triplicate. When the cells reached 90% confluence, one artificial wound was scratched with a sterile 20 µL pipette tip to generate a uniform wound. The medium was then discarded, and cells were washed twice with PBS to remove debris. The wound closure was monitored, and photographs were taken by an Olympus microscope at 24 h post wounding. 

### 4.11. Cell Migration Assay

Cell migration assay was performed in 24-well Transwell plates with 8.0 µm pore polycarbonate membrane insert (Corning). Non-transfected, scrambled-, PD-L1- or CD73- siRNA transfected cells were seeded in the upper chambers at 2 × 10^4^ cells per insert in 100 µL medium containing 1% FBS. The bottom chambers were filled with 650 µL medium with 10% FBS. After 24 h incubation, the inserts were transferred into a new 24-well plate, fixed with PFA and stained with crystal violet. Non-migrated cells in the upper inserts were removed carefully using cotton swabs and the migrated cells were then photographed using an Olympus microscope. 

### 4.12. Statistical Analysis

All values in the figures and text were shown as means ± SD. Statistical analyses were performed using the GraphPad Prism 8 statistical software package. Any significant differences among mean values were evaluated by the Student’s *t*-test. A two-sided *p* < 0.05 was accepted as significant, or as indicated in the figures/legends.

## Data Availability

All datasets generated for this study are included in the article/supplementary material.

## Supplementary Materials

The following supporting information can be downloaded at: https://www.mdpi.com/article/10.3390/ijms23031565/s1.
