# Supplementary material for: CD73 and PD-L1 as Potential Therapeutic Targets in Gallbladder Cancer
Source: Int J Mol Sci. 2022 Jan 29;23(3):1565. doi: 10.3390/ijms23031565 (PMC8836068; doi:10.3390/ijms23031565)
Supplement: Supplementary file 1 [file ijms-23-01565-s001.zip › ijms-1516038-supplementary.pdf]

Table S1. List of primers for RT-qPCR

Gene Name	Forward Sequence (5′-3′)	Reverse Sequence (5′-3′)
ABCG2	GTTCTCAGCAGCTCTTCGGCTT	TCCTCCAGACACACCACGGATA
CD73	AGTCCACTGGAGAGTTCCTGCA	TGAGAGGGTCATAACTGGGCAC
VISTA	AGATGCACCATCCAACTGTGTGG	AGGCAGAGGATTCCTACGATGC
HVEM	TTCTCTCAGGGAGCCTCGTCAT	CTCACCTTCTGCCTCCTGTCTT
PD1	GGTGACAGAGAGAAGGGCAG	GTCCTCCTTCAGGGGCTGG
TIGIT	TGGTGGTCATCTGCACAGCAGT	TTTCTCCTGAGGTCACCTTCCAC
TIM3	CTACTGCTGCCGGATCCAAA	GTCCCCTGGTGGTAAGCATC

Table S2. List of antibodies used for western blot.

Antibodies	Cat. No.	Manufacturer	Antibody Category	Dilution
CD44	ab157107	Abcam	Primary	1:10000
β-actin	4967s	Cell signaling	Primary	1:4000
CD24	PA5-86955	Thermofisher	Primary	1:500
ALDH1A1	sc-374194	Santa Cruz	Primary	1:500
E-CAD	ab76055	Abcam	Primary	1:1000
N-CAD	sc-53488	Santa Cruz	Primary	1:500
OCCLUDIN	sc-133255	Santa Cruz	Primary	1:200
FIBRONECTIN	sc-8422	Santa Cruz	Primary	1:200
ZEB1	sc-25388	Santa Cruz	Primary	1:250
VIMENTIN	ab92547	Abcam	Primary	1:2000
SLUG	sc-166902	Santa Cruz	Primary	1:200
PDL1	ab238697	Abcam	Primary	1:1000
GALECTIN9	ab227046	Abcam	Primary	1:1000
TIM3	ab241332	Abcam	Primary	1:1000
GAPDH	MAB376	Millipore	Primary	1:100,0000

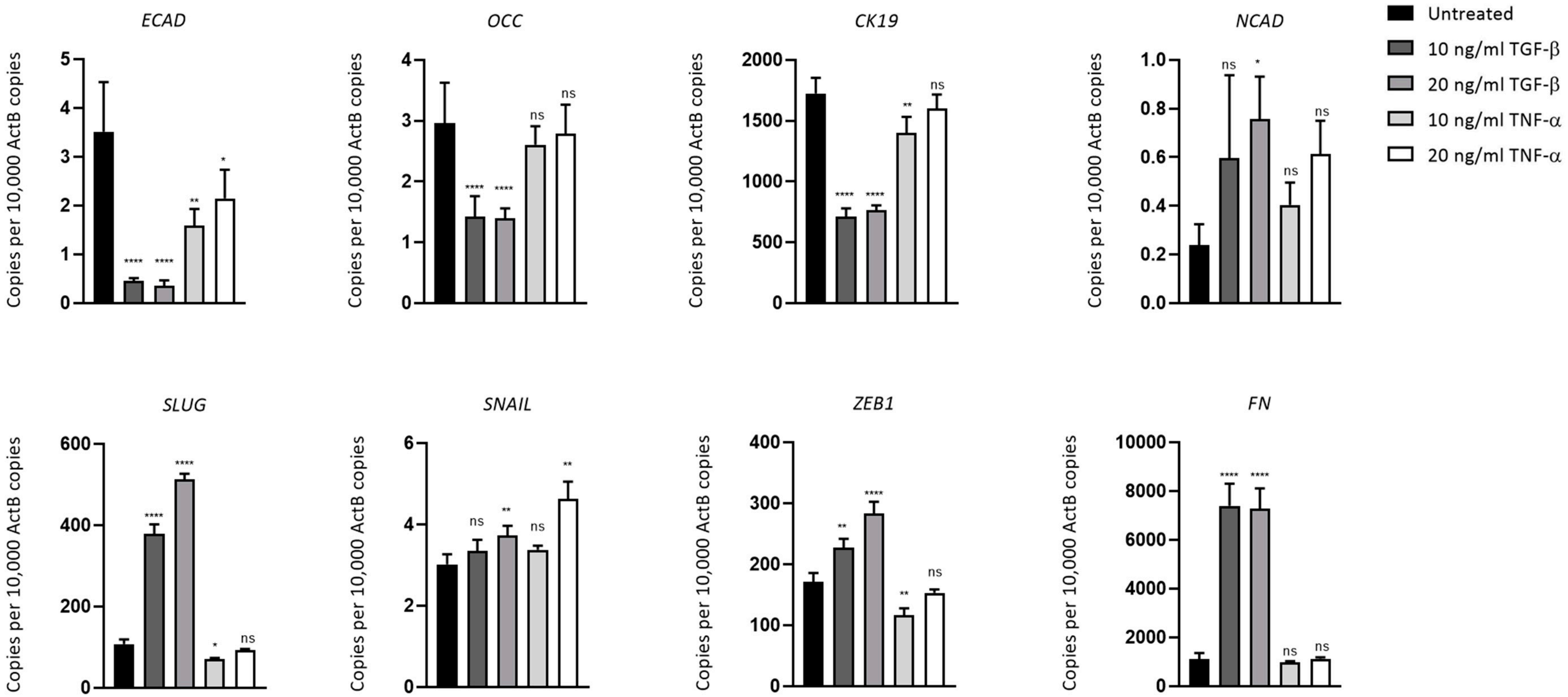

Figure S1. Incubation with TGF-β1 or TNF-α for 96 hrs induced EMT to varied extend in NOZ cells. ns not significant, *P<0.05, **P<0.01, ****P<0.001.

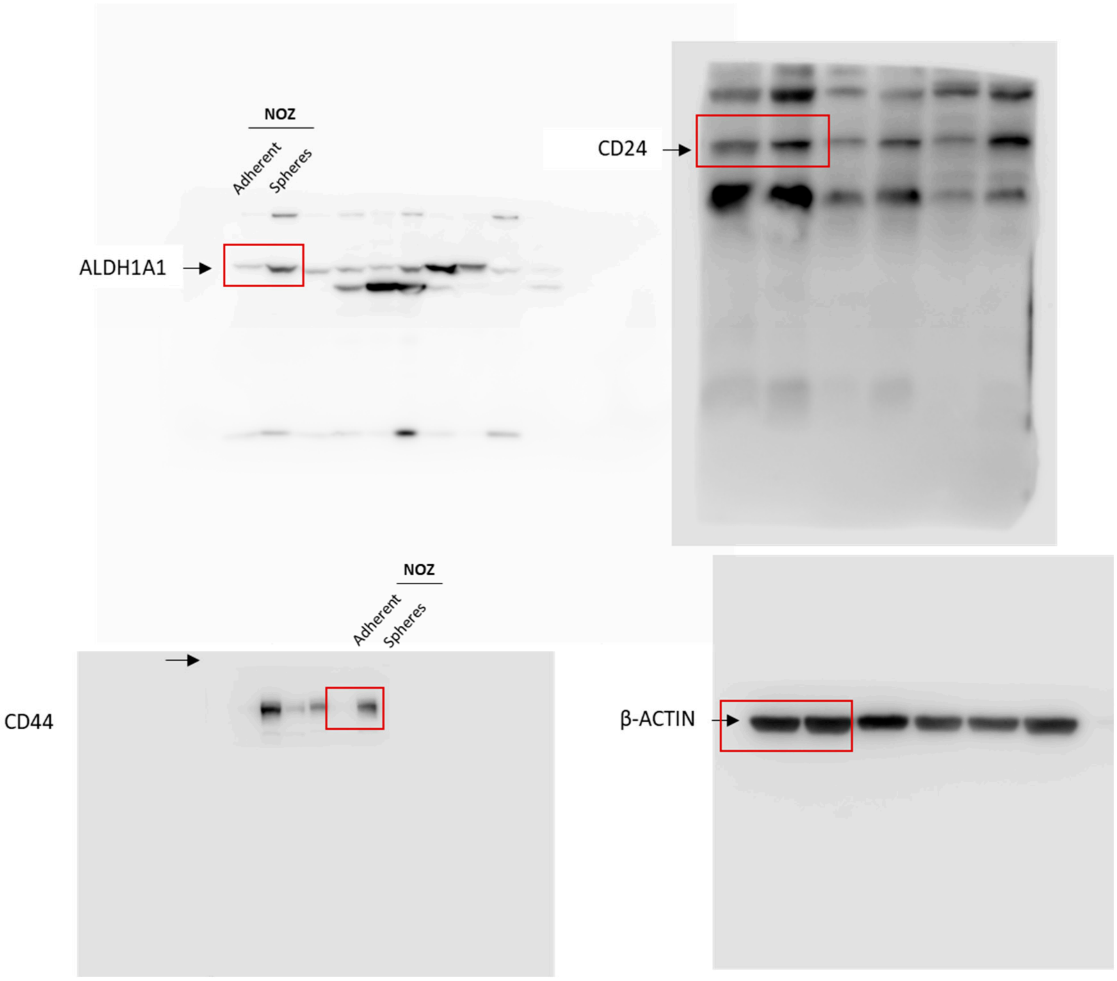

Figure S2. Original western blots from figure 1C.

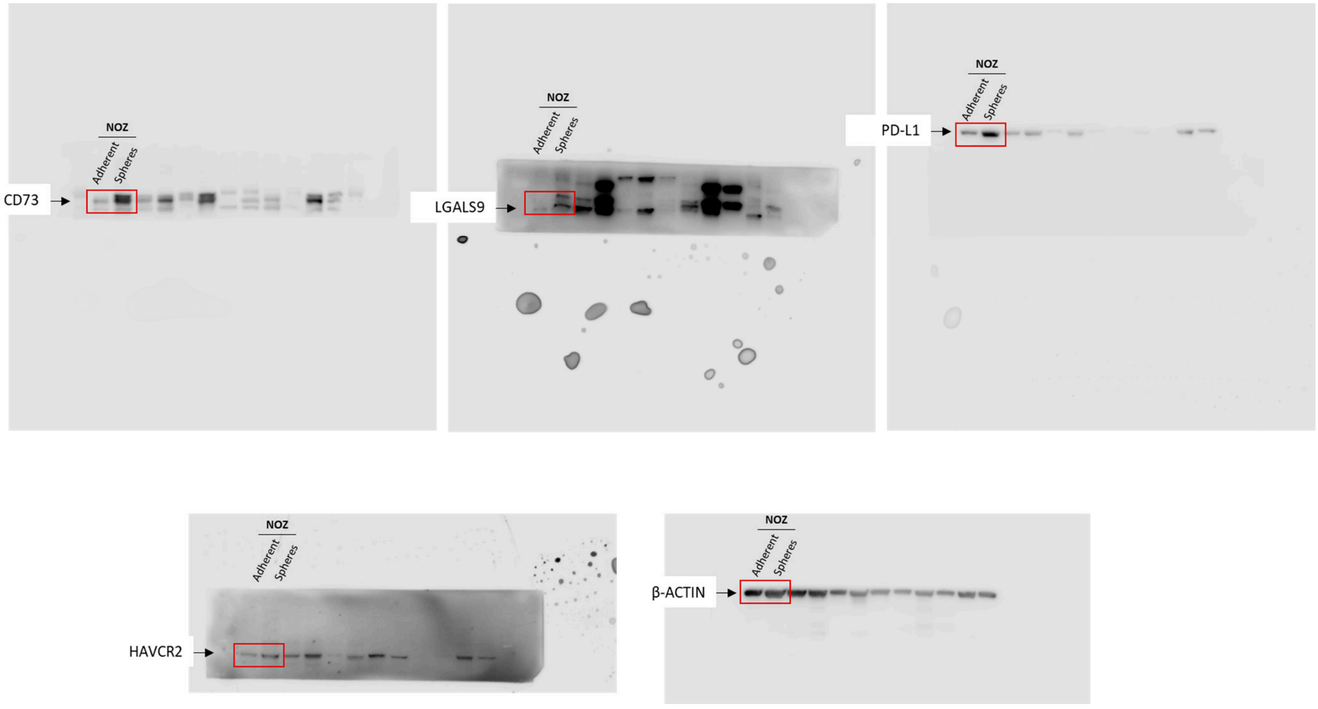

Figure S3. Original western blots from figure 2B.

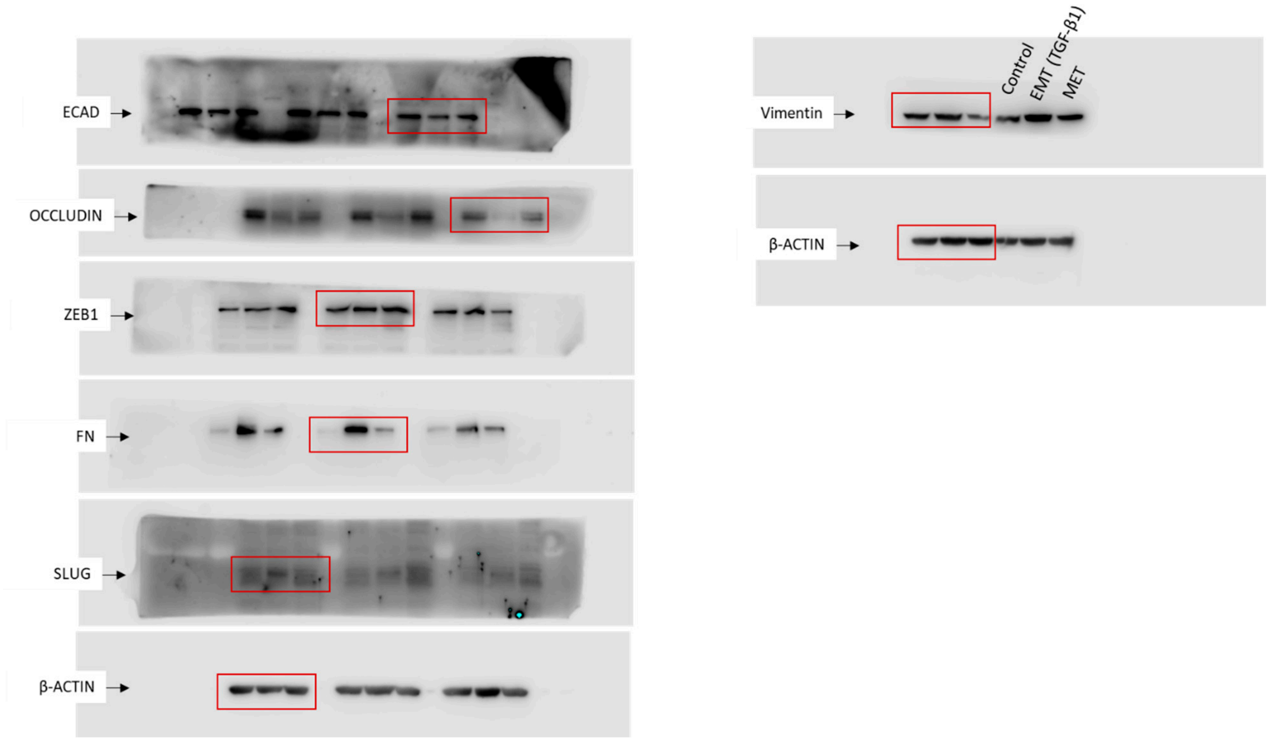

Figure S4. Original western blots from figure 4B.

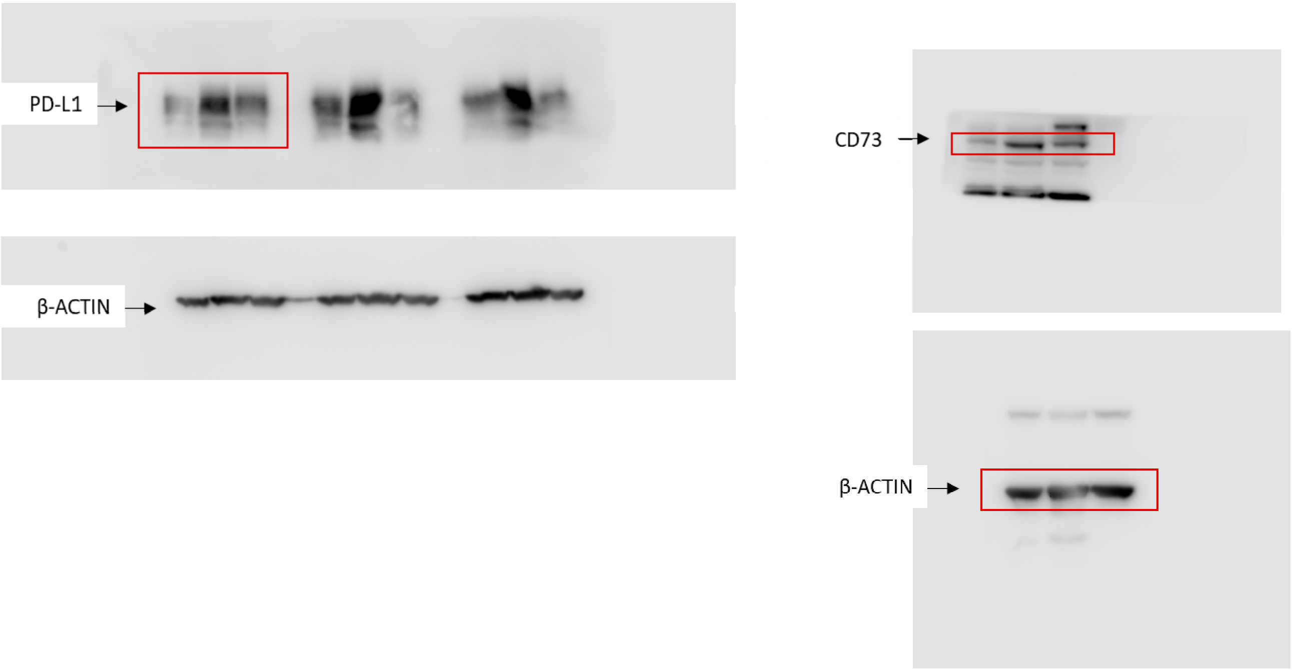

Figure S5. Original western blots from figure 5B.

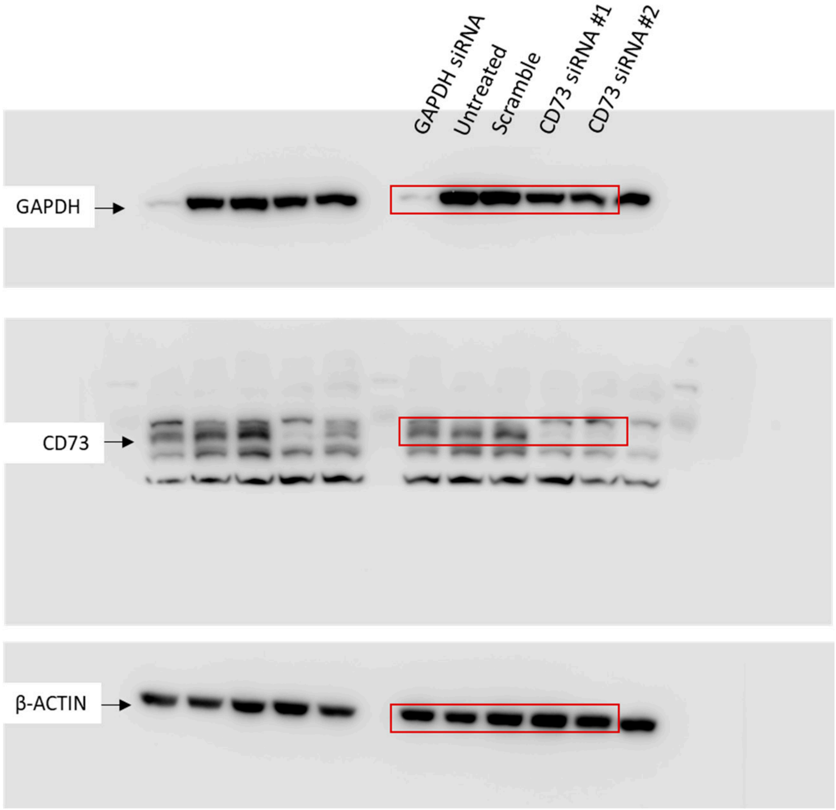

Figure S6. Original western blots from figure 6B.

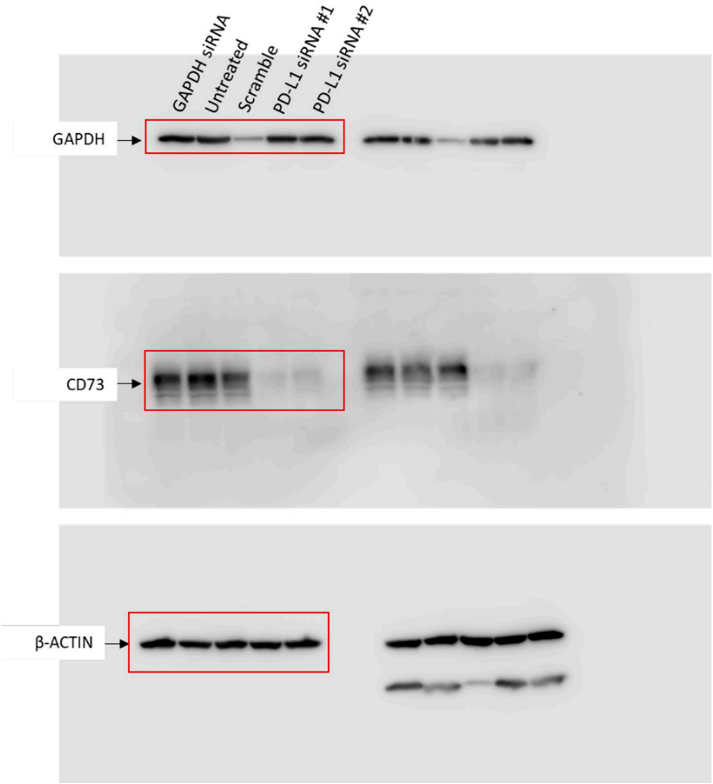

Figure S7. Original western blots from figure 6D.
